# Prevalence and characteristics of antibiotic prescription for acute COVID-19 patients in Japan

**DOI:** 10.1038/s41598-022-26780-0

**Published:** 2022-12-26

**Authors:** Seiji Hamada, Yasuharu Tokuda, Hitoshi Honda, Takashi Watari, Tomoharu Suzuki, Takuhiro Moromizato, Masashi Narita, Kiyosu Taniguchi, Kenji Shibuya

**Affiliations:** 1 Urasoe General Hospital, Urasoe, Okinawa Japan; 2 The Tokyo Foundation for Policy Research, Minato-ku, Tokyo, Japan; 3 Muribushi Okinawa Center for Teaching Hospitals, 3-42-8 Iso, Urasoe, Okinawa 901-2132 Japan; 4 Fujita Medical University, Toyoake, Aichi Japan; 5 General Medicine Center, Shimane University, Matsue, Shimane Japan; 6 Okinawa Prefectural Nanbu Medical Center & Children’s Medical Center, Haebaru, Okinawa Japan; 7 National Hospital Organization Mie National Hospital, Tsu, Mie Japan; 8 University of Tsukuba School of Medicine, Tsukuba, Japan

**Keywords:** Epidemiology, Infectious diseases

## Abstract

COVID-19 is a viral infection and does not require antibiotics. The study aimed to elucidate a prescribing pattern of antibiotics for COVID-19. A nationwide cross-sectional study was conducted in Japan. The Diagnosis and Procedure Combinations (DPC) data was used to collect information, covering 25% of all acute care hospitals in the country. In 140,439 COVID-19 patients, 18,550 (13.21%) patients received antibiotics. Antibiotics were prescribed more often in inpatients (10,809 out of 66,912, 16.15%) than outpatients (7741 out of 73,527, 10.53%) (*p* < 0.001). Outpatient prescription was significantly associated with older patients (odds ratio [OR], 4.66; 95% confidence interval [CI] 4.41–4.93) and a greater Charlson index (OR with one-point index increase, 1.22; 95% CI 1.21–1.23). Inpatient prescription was significantly associated with older patients (OR 2.10; 95% CI 2.01–2.21), male gender (OR 1.12, 95% CI 1.07–1.18), a greater Charlson index (OR with one-point increase, 1.06; 95% CI 1.05–1.07), requirement of oxygen therapy (OR 3.44; 95% CI 3.28–3.60) and mechanical ventilation (OR 15.09; 95% CI 13.60–16.74). The most frequently prescribed antibiotic among outpatients was cefazolin, while that among inpatients was ceftriaxone. Antibiotic prescription is relatively low for acute COVID-19 in Japan. Antibiotic prescription was associated with older age, multi-morbidity, severe disease, and winter season.

## Introduction

Antibiotic prescription for viral infection without bacterial coinfection or secondary infection is low-value care because it provides no beneficial pharmacological effect on viral syndrome^1–4^. Additionally, unnecessary antibiotic prescriptions may lead to the spread of antimicrobial resistance, causing a threat to humanity^5^. In the mid-2010s, many countries, including Japan, announced a National Action Plan to address antimicrobial resistance by reducing inappropriate antibiotic prescription^6,7^. However, acute COVID-19 can cause potentially fatal infection and thus, if the diagnosis is not certain due to a delayed turnaround time of nucleic acid testing, a false-negative test result, a false-positive sputum Gram stain, or a false-positive urine bacterial antigen, empiric antibiotic use can be reasonable.

Several studies have been conducted regarding prevalence of antibiotic prescription and characteristics of acute COVID-19 patients associated with antibiotic prescription^8–11^. In a study examining the first year of the pandemic in the US^12^, about 30% of older outpatients with acute COVID-19 received antibiotic prescriptions with male gender and winter season related to a higher rate of prescription. However, there have been few studies which examined prevalence and characteristics of nationwide antibiotic prescription in Japan. In addition, the association between prescription and comorbidity or disease severity has been unclear. If the prescription prevalence is higher in Japan, educational intervention may be implemented to improve prescription practice in Japan. Elucidation of the prescription characteristics may be helpful to address the prescription practice. Thus, our study aimed to examine prevalence and characteristics of antibiotic prescription using a large administrative database in Japan.

## Methods

### Data collection and patients

Our study used a prospectively registered nationwide hospital administrative database, called diagnosis and procedure combination (DPC), to collect data from many acute care hospitals (DPC hospitals). The government of Japan provides advantages in reimbursement payment to DPC hospitals, thus the majority of middle to large-sized hospitals are part of the DPC register. Regarding DPC data used in our study, 438 of the approximately 1750 DPC hospitals in Japan provided data, which is around 25% coverage, through the Medical Data Vision (MDV), Co., Tokyo, Japan (As of April 1, 2021)^13^. The company covers DPC hospitals nationwide, and the age and gender distribution of registered patients are comparable to that of patient data of healthcare institutions nationwide as published by the Ministry of Health, Labour and Welfare, thus is a fair representation of the national data. The data provided to the MDV did not include information that could be used to identify individual patients such as address, race, and prefectural area because of the confidentiality contract between each hospital and the MDV. Around 10% of the hospitals provided data on outpatients to the MDV. We analyzed DPC data for patient demographics (age and gender), diagnoses, comorbidity, prescriptions, and procedures. DPC data of the MDV have been used in recent studies on COVID-19^14,15^.

Our study collected data from 1 January 2020 to 30 November 2021 and focused on patients with confirmed COVID-19 (International Classification of Diseases and Related Health Problems, 10th Revision diagnosis code U071) in both the outpatient and inpatient setting in all age ranges. Acute COVID-19 patients who were initially seen in the outpatient setting but were subsequently required for admission were classified as inpatients. Information on antibiotics was collected if prescribed within 7 days before or after diagnosis of COVID-19^12,16^.

Data was collected for the number of cases of acute COVID-19 diagnosis and the number of prescriptions by month, including highly prescribed antibiotics. Information on prescribing physicians’ characteristics (specialty, age, gender) in the DPC database was unavailable. Our study was approved by the Muribushi Okinawa Ethics Committee (No. 2021-9). All methods were performed in accordance with the relevant guidelines and regulations by including a statement in the methods section. The study was a retrospective study using the administrative anonymized data and therefore the informed consent of individual patients was waived.

#### Patient and public involvement statement

Patients or the public were not involved in the design, or conduct, or reporting, or dissemination plans of our research.

### Statistical analyses

Regarding descriptive data analysis, numeric variables were described as mean and standard deviation, while nominal variables were described as count and proportion. The prevalence of antibiotic prescriptions was compared between inpatients and outpatients using Fisher’s exact test. Regarding multivariable data analysis, logistic regression analysis was used to examine the association between antibiotic prescription and age, gender, and Charlson comorbidity index in outpatient settings. To calculate Charlson index of comorbidity, we used algorithms for defining comorbidities using administrative data in ICD-9-CM and ICD-10 based on Quan et al.^17^. Similarly, logistic regression analysis was used for examining the association between antibiotic prescription and age, gender, Charlson comorbidity index and COVID-19 severity indicators (requiring oxygen therapy or invasive mechanical ventilation within 7 days after admission date) in inpatient settings. Analysis was conducted using STATA version 16 (NC, US). P-value less than 0.05 was defined as statistically significant.

## Results

We examined a total of 140,439 COVID-19 patients (mean age ± standard deviation, 47.39 ± 22.90; male, 55.84%). Of these patients, 66,912 patients (47.64%) were admitted and were considered inpatients, while the remaining 73,527 were considered outpatients. Altogether, 18,550 (13.21%) patients of 140,439 COVID-19 patients were prescribed antibiotics. Inpatients were more likely to be prescribed antibiotics with 10,809 (16.15%) of 66,912 patients compared to 7741 (10.53%) of 73,527 in outpatients (*p* < 0.001). There were 13,350 older outpatients and 4271 (31.99%) of them were prescribed antibiotics, while 5885 (26.44%) of 22,260 older inpatients were prescribed antibiotics.

Regarding patient characteristics as a possible factor for prescriptions in outpatients (Table 1A), antibiotic prescription was significantly associated with older patients (odds ratio [OR] 4.66; 95% confidence interval [CI] 4.41–4.93) and a greater Charlson index score (OR with one-point index increase, 1.22; 95% CI 1.21–1.23). Regarding patient characteristics for prescriptions in inpatients (Table 1B), antibiotic prescription was significantly associated with the older patients (OR 2.10; 95% CI 2.01–2.21), male gender (OR 1.12, 95% CI 1.07–1.18), a greater Charlson index score (OR with one-point index increase, 1.06; 95% CI 1.05–1.07), oxygen therapy requirement (OR 3.44; 95% CI 3.28–3.60) and requirement for mechanical ventilation (OR 15.09; 95% CI 13.60–16.74). Among inpatients, in-hospital mortality was higher in the prescribed group at 12.33% (1332/10,809) than in the non-prescribed group at 1.89% (1058/56,103). Also, among inpatients, mean hospital stay was longer in the prescribed group at 17.42 days (SD 0.17) than in the non-prescribed group at 10.79 days (SD 0.04).

**Table 1:** Patient characteristics for antibiotic prescription and outcomes among outpatients or inpatients.

Characteristic	Antibiotics prescribed (n = 7741)	Not prescribed (n = 65,786)	Adjusted odds ratio (95% CI)* for antibiotic prescription
**A: Outpatients with acute COVID-19 between Jan 2020 and Nov 2021 in Japan (N = 73,527)**			
Older patients, n (%)	4271 (55.17%)	9079 (13.80%)	4.66 (4.41, 4.93)
Male, n (%)	4308 (55.65%)	35,667 (54.22%)	1.02 (0.97, 1.08)
Charlson index, mean (SD)	3.09 (3.51)	0.71 (1.82)	1.22 (1.21, 1.23) ***

Characteristic/outcome	Antibiotics prescribed (n = 10,809)	Not prescribed (n = 56,103)	Adjusted odds ratio (95% CI)** for antibiotic prescription
**B: Inpatients with acute COVID-19 between Jan 2020 and Nov 2021 in Japan (N = 66,912)**			
Older patients, n (%)	5885 (54.45%)	16,375 (29.19%)	2.10 (2.01, 2.21)
Male, n (%)	6690 (61.89%)	31,758 (56.61%)	1.12 (1.07, 1.18)
Charlson index, mean (SD)	3.59 (3.11)	2.34 (2.81)	1.06 (1.05, 1.07)***
Oxygen therapy, n (%)	6596 (61.02)	15,073 (26.87)	3.44 (3.28, 3.60)
Invasive mechanical ventilation, n (%)	1674 (15.49)	564 (1.01)	15.09 (13.60, 16.74)
In-hospital mortality, n (%)	1332/10,809 (12.33%)	1058/56,103 (1.89%)	N/A
Hospital stay, mean days (SD)	17.42 (0.17)	10.79 (0.04)	N/A

*SD* standard deviation, *CI* confidence interval.

*Adjusted for older patients, male, Charlson index.

**Adjusted for older patients, male, Charlson index, requiring oxygen therapy, and requiring invasive mechanical ventilation. Odds ratios for antibiotic prescription are not estimated for in-hospital mortality and hospital stay, because these outcomes are not considered as predictors for antibiotic prescription.

***Odds ratio by one point increase of the index score.

Table 2 shows the most frequently prescribed antibiotics by ranking. The most frequently prescribed antibiotics among outpatients were cefazolin (n = 1446), ceftriaxone (943), and ampicillin/sulbactam (817). The most frequently prescribed antibiotics among inpatients were ceftriaxone (n = 3502), ampicillin/sulbactam (1500), and azithromycin (1369).

**Table 2:** Frequently prescribed antibiotics.

A. Outpatients			B. Inpatients		
Rank	Antibiotic	Patients	Rank	Antibiotic	Patients
1	Cefazolin	1446	1	Ceftriaxone	3502
2	Ceftriaxone	943	2	Ampicillin/Sulbactam	1500
3	Ampicillin/Sulbactam	817	3	Azithromycin	1369
4	Levofloxacin	663	4	Levofloxacin	1093
5	Cefmetazole	532	5	Piperacillin/Tazobactam	897
6	Azithromycin	472	6	Meropenem	363
7	Piperacillin/Tazobactam	402	7	Clarithromycin	316
8	Clarithromycin	289	8	Garenoxacin	228
9	Amoxicillin	288	9	Cefepime	150
10	Meropenem	206	10	Amoxicillin	144

**Figure 1:**
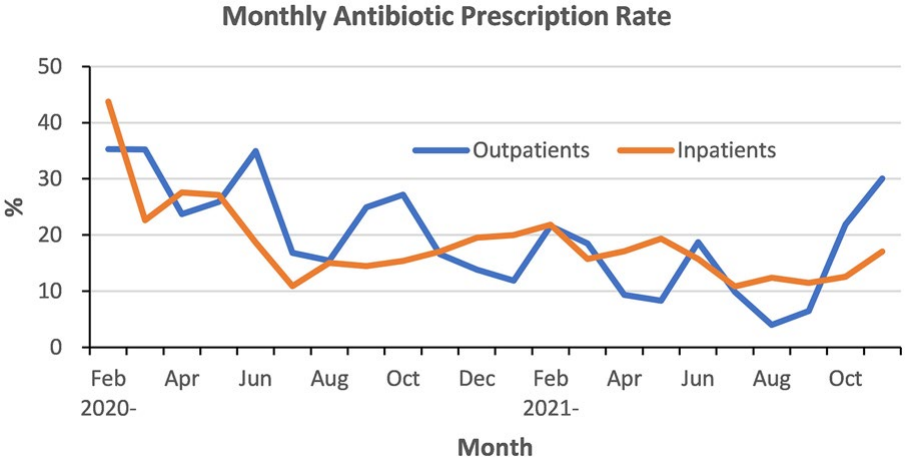
Monthly prevalence of antibiotic prescription between February 2020 and November 2021. The data of Jan 2020 is omitted because of the low number of patients (outpatient, n = 2; inpatient, n = 0).

The monthly antibiotic prescription rate is shown in Fig. 1. The first case of COVID-19 was reported on 16 January 2020 and the number of cases was relatively small in January 2020, and thus the rate was not described in that month. The highest prescription rate is in the first winter (February 2020) and the lowest in the summer season, with the following range: 4% in August 2021 to 35.3% in February 2020 for outpatients; 10.9% in July 2020 and 2021 to 43.8% in February 2020 for inpatients.

## Discussion

Our study suggests that the overall prescription rate of antibiotics among acute COVID-19 patients including outpatients and inpatients in Japan was around 13%. The rate was higher in winter and was associated with patient characteristics including older age, multi-morbidity, and severe disease requiring oxygen or invasive mechanical ventilation. Higher mortality and longer hospital stay in the prescribed inpatient group were likely to reflect that the prescribed group had severe disease. Although older age was a significant factor associated with prescription, older outpatients were more likely to receive antibiotics than older inpatients. Frequently used antibiotics included cephalosporins and penicillin with beta-lactamase inhibitors. To our knowledge, this may be the first study to determine prevalence and patient characteristics associated with antibiotic prescription in a large population with COVID-19.

In our study, the antibiotic prescription rate in COVID-19 patients was lower than the prescription rate in patients with upper respiratory symptoms in Japan prior to the COVID-19 pandemic (up to 20%)^18^, as well as in the US^16^. Although 38% of Japanese physicians in a national survey reported that they had clinical experience of prescribing antibiotics to patients with the common cold in outpatient clinics in the period before the pandemic, 96% of them recognized that they tried to follow the antibiotic stewardship program over the last year before the survey^18^. However, some patients with acute COVID-19 may have bacterial co-infection or secondary bacterial infection. A study noted that approximately eight percent of patients with acute COVID-19 who visited the emergency departments had presumed bacterial infection based on the finding of cultures growing clinically important bacteria at their ER visit^9^. In a meta-analysis^19^ based on data on acute COVID-19 up to April 2020, bacterial co-infection was identified in 3.5% of patients based on estimates on presentation and secondary bacterial infection in 14.3% of patients, but 73% of patients analyzed in this meta-analysis received antibiotics. Moreover, several studies which were published later, revealed that bacterial co-infection or secondary bacterial infection was noted in 1.2%^20^, 2.3%^21^ and 6.1%^22^ of all patients with acute COVID-19. When considering the low proportion of bacterial co-infection or secondary bacterial infection among patients with acute COVID-19, 13% of antibiotic prescriptions in patients with COVID-19 still may need improvement. Although a study outside Japan reported that physicians tend not to follow guideline statements against antibiotic prescription among patients with acute viral infections^23^, physicians in Japan need to address the prescription practice by safely reducing its use since patients with acute COVID-19 are likely to have a low probability of bacterial coinfection^24^.

The proportion among older outpatients with COVID-19 accounted for 32.0%, which was higher than that among older inpatients in our study. The US study also showed a proportion of around 30% among older outpatients, which is comparable to the result among older outpatients of our study^12^. Since admission is usually feasible for patients with severe diseases in Japan, and thus outpatients had less severe diseases than inpatients, it is unclear why the older outpatients in our study received more antibiotic prescriptions than the older inpatients. During the upsurge of COVID-19, a high proportion of patients with COVID-19 may have received antibiotics at outpatient settings even if they were indicated for admission, due to the high bed occupancy rate. Moreover, diagnostic uncertainty about COVID-19 might have been greater among outpatients than inpatients because of outpatients’ limited availability of testing to rule in COVID-19 as well as to rule out other possible causes of infectious disease.

In line with findings in a US study, the higher rate of antibiotic prescriptions in winter compared to summer was revealed by our monthly data^12,25^. Physicians examining patients with respiratory symptoms in winter might have a higher tendency to suspect bacterial pneumonia. Indeed, winter has a higher incidence of bacterial pneumonia and common respiratory viral infections^25,26^. Coinfection of bacteria and viruses, including SARS-CoV2, may occur, however, cases of COVID-19 with coinfection of bacterial pneumonia have been reported in mostly late-stage ventilator-associated pneumonia and rarely early-stage community-acquired bacterial pneumonia^27,28^. The prescription rate in the second winter (year of 2021) was lower than that of the first winter (year of 2020) in our study. This might have reflected the improvement of timely COVID-19 diagnosis as diagnostics gradually became available after the initial waves of the pandemic.

Bacterial coinfection such as pneumonia by *Streptococcus pneumoniae* or *Staphylococcus aureus* with COVID-19 was lower than those with influenza^29^. In a 10-year retrospective cohort study comparing bacterial co-infection in patients admitted with either COVID-19, influenza or RSV (respiratory syncytial virus) positive community-acquired pneumonia^29^, bacterial coinfection was identified in only four percent (46/1243) of COVID-19 patients who were observed between 2020 and 2021. In contrast, the coinfection was identified in 27% (209/775) of those with influenza between 2010 and 2021. In a comparison of outpatient antibiotic use between COVID-19 and influenza, a study in the U.S. showed that approximately one third of patients with PCR-confirmed influenza were prescribed antibiotics and that patients who received antibiotics were relatively older.

The findings of our study that older age, multi-morbidity, and severe COVID-19 requiring oxygen or invasive mechanical ventilation are associated with antibiotic prescription may reflect that the treatment threshold for antibiotic prescription is changed based on individual risk^30^. For example, physicians more frequently tried to manage patients with severe COVID-19 with possible treatable bacterial infection or those with high risk for severe disease (older patients or those with multi-morbidity). A large study on COVID-19 inpatients in Japan during the first to third waves showed that about 5% developed bacterial pneumonia and the risk was higher in older patients^31^. In the usual clinical course of COVID-19 patients, secondary bacterial infection may develop, if any, several days after onset and thus routine antibiotic prescription at the time of COVID-19 diagnosis is not recommended^3,4^. Nonetheless, there may be an opportunity to improve the antibiotic prescription practice by enhancing diagnostic excellence to aim for greater clarity of the threshold to commencing antibiotic treatment in individual patients.

The type of antibiotics prescribed in COVID-19 patients in our study differed from that of the US study on outpatients, with the highest prescribed being beta-lactams in the former and azithromycin (51%) in the latter^12^. The choice of azithromycin in US physicians might be related to the influence of several studies indicating the possible usefulness of azithromycin among acute COVID-19 patients^32,33^. In a survey study on physicians and their antibiotic prescription pattern for patients with suspected acute respiratory tract infection in Japan^18^, beta-lactams or beta-lactams/beta-lactamase inhibitors (52%) were prescribed more often than macrolides (35%) or fluoroquinolones (11%). Several antibiotics, such as cefazolin, ceftriaxone, and ampicillin/sulbactam, are available only for parenteral use. A potential explanation for the overuse of cefazolin or other antibiotics for community-acquired infections is the presence of a wide variety of bacterial co-infection in patients with COVID-19. A study suggested that bacterial co-infection in these patients may be of an etiology independent of COVID-19, commonly occurring in the elderly population. The presence of these infections possibly led to empirical overuse of these agents^34^, although the usefulness of antibiotics for the fatal cases remains unclear.

There are several limitations in our study. First, we could not adjust the confounding factors and draw a causal inference regarding antibiotic prescribing patterns due to the cross-sectional study design. Second, we could not exclude cases of concomitant bacterial infections of the lungs or other organ systems because of the lack of clinical reasons for the prescription in individual patients in the database. Moreover, we could not obtain clinical data of individual patients about the history and physical examination, culture results (sputum, blood, urine, and/or cerebrospinal fluid cultures), procalcitonin level, leukocyte count, and/or C-reactive protein level which physicians used to evaluate the probability of bacterial infection. Third, the participating hospitals that consented to data provision in the database were not randomly sampled. However, the database was large, and the distribution of demographics, diagnoses, and procedures was extremely similar to the national survey data, thus the practice patterns are likely to be similar across all Japanese acute care hospitals. Fourth, we could not collect antibiotic prescribing data in other types of healthcare institutions, including clinics, non-acute care hospitals, and geriatric long-term care facilities. Patients treated in these institutions may have different patterns in receiving prescriptions, which warrants further studies.

In conclusion, the antibiotic prescription rate among acute COVID-19 patients, including outpatients and inpatients, in Japan was relatively low, but room for improvement in antibiotic prescribing practice remains. The higher rate was recognized in winter and was associated with older age, multi-morbidity, and severe disease. In addition to the diagnostic uncertainty of COVID-19, possible bacterial coinfection and secondary bacterial infection may have caused physicians to prescribe antibiotics. Nevertheless, judicious use of antibiotics in patients with COVID-19 should be strongly encouraged even if the COVID-19 pandemic continues. Early detection of bacterial coinfection or secondary bacterial infection with wise use of bacterial cultures and inflammatory biomarkers should improve clinical management for COVID-19 patients.

## Data Availability

All data relevant to the study are owned by the MDV, Tokyo, Japan, and the corresponding author can be contacted for requesting the data availability.
